# Maternal exposure to polyethylene micro- and nanoplastics impairs umbilical blood flow but not fetal growth in pregnant mice

**DOI:** 10.1038/s41598-023-50781-2

**Published:** 2024-01-03

**Authors:** Jenna Hanrahan, Katherine L. Steeves, Drew P. Locke, Thomas M. O’Brien, Alexandre S. Maekawa, Roshanak Amiri, Christopher K. Macgowan, Ahmet A. Baschat, John C. Kingdom, André J. Simpson, Myrna J. Simpson, John G. Sled, Karl J. Jobst, Lindsay S. Cahill

**Affiliations:** 1 Department of Chemistry, Memorial University of Newfoundland, Arctic Avenue, St. John’s, NL A1C 5S7 Canada; 2 Translational Medicine, Hospital for Sick Children, Toronto, ON M5G 1X8 Canada; 3 Department of Medical Biophysics, University of Toronto, Toronto, ON M5G 1L7 Canada; 4 Department of Gynecology and Obstetrics, Johns Hopkins Center for Fetal Therapy, Johns Hopkins University, Baltimore, MD 21287 USA; 5 Department of Obstetrics and Gynecology, University of Toronto, Toronto, ON M5G 1E2 Canada; 6 Mount Sinai Hospital, Toronto, ON M5G 1X5 Canada; 7 Environmental NMR Centre and Department of Physical and Environmental Sciences, University of Toronto, Toronto, ON M1C 1A4 Canada; 8 Mouse Imaging Centre, Hospital for Sick Children, Toronto, ON M5T 3H7 Canada; 9 Discipline of Radiology, Memorial University of Newfoundland, St. John’s, NL A1C 5S7 Canada

**Keywords:** Environmental sciences, Intrauterine growth

## Abstract

While microplastics have been recently detected in human blood and the placenta, their impact on human health is not well understood. Using a mouse model of environmental exposure during pregnancy, our group has previously reported that exposure to polystyrene micro- and nanoplastics throughout gestation results in fetal growth restriction. While polystyrene is environmentally relevant, polyethylene is the most widely produced plastic and amongst the most commonly detected microplastic in drinking water and human blood. In this study, we investigated the effect of maternal exposure to polyethylene micro- and nanoplastics on fetal growth and placental function. Healthy, pregnant CD-1 dams were divided into three groups: 10^6^ ng/L of 740–4990 nm polyethylene with surfactant in drinking water (*n* = 12), surfactant alone in drinking water (*n* = 12) or regular filtered drinking water (*n* = 11). At embryonic day 17.5, high-frequency ultrasound was used to investigate the placental and fetal hemodynamic responses following exposure. While maternal exposure to polyethylene did not impact fetal growth, there was a significant effect on placental function with a 43% increase in umbilical artery blood flow in the polyethylene group compared to controls (p < 0.01). These results suggest polyethylene has the potential to cause adverse pregnancy outcomes through abnormal placental function.

## Introduction

Plastics are ubiquitous in our indoor and outdoor environments. Following physical and photo-oxidative degradation, they break down into smaller pieces that are therefore more mobile. Plastic particles derived via breakdown that have a diameter less than 5 mm are known as microplastics^1^ while nanoplastics are those with a diameter less than 1 μm^2,3^. Micro- and nanoplastics are known to be found in the open ocean where marine organisms are readily exposed, leading to accumulation through the higher levels of the food chain to humans^4^. Another route of human exposure to micro- and nanoplastics is through tap and bottled drinking water. A recent review reported that microplastics are present in drinking water in concentrations up to 10^4^ particles/L^5^. The most common microplastic detected thus far is polyethylene, consistent with polyethylene having the highest annual production rate of any plastic worldwide^6^. This widespread presence and dispersion of plastics into the global environment raises concerns about their impact on human health and reproduction.

Micro- and nanoplastics have been reported in adult human blood, with polyethylene terephthalate, polyethylene and polymers of styrene at the highest concentrations^7^. Most concerning for pregnancy and fetal development, microplastics have been found in human placental tissue^8–10^ and in fetal meconium^11,12^. To explore the direct maternal impact of micro- and nanoplastics, exposure studies have been conducted using experimental mice, a well-accepted animal model of pregnancy^13^. Maternal exposure to polystyrene particles results in fetal growth restriction in mice^14,15^, altered placental metabolism^16^, metabolic disorders in the offspring^17,18^, disturbances in the maternal–fetal immune system^19^, structural abnormalities in the brain of the offspring^20^, and abnormal placental blood flow^21^. A gap in the plastics research so far is that the majority of animal studies on the impact of plastics exposure have used polystyrene particles. While the use of polystyrene is environmentally relevant, the prevalence of polyethylene in our environment motivates study of this common polymer. A recent study investigated exposure to polyethylene microplastics (10–20 μm) during pregnancy, finding that the mice offspring demonstrated traits consistent with autism spectrum^22^.

In the present study, we investigated the effect of maternal exposure to polyethylene plastics on placental size, fetal growth and feto-placental blood flow using experimental mice and whether these effects were dependent on fetal sex.

## Results

**Figure 1:**
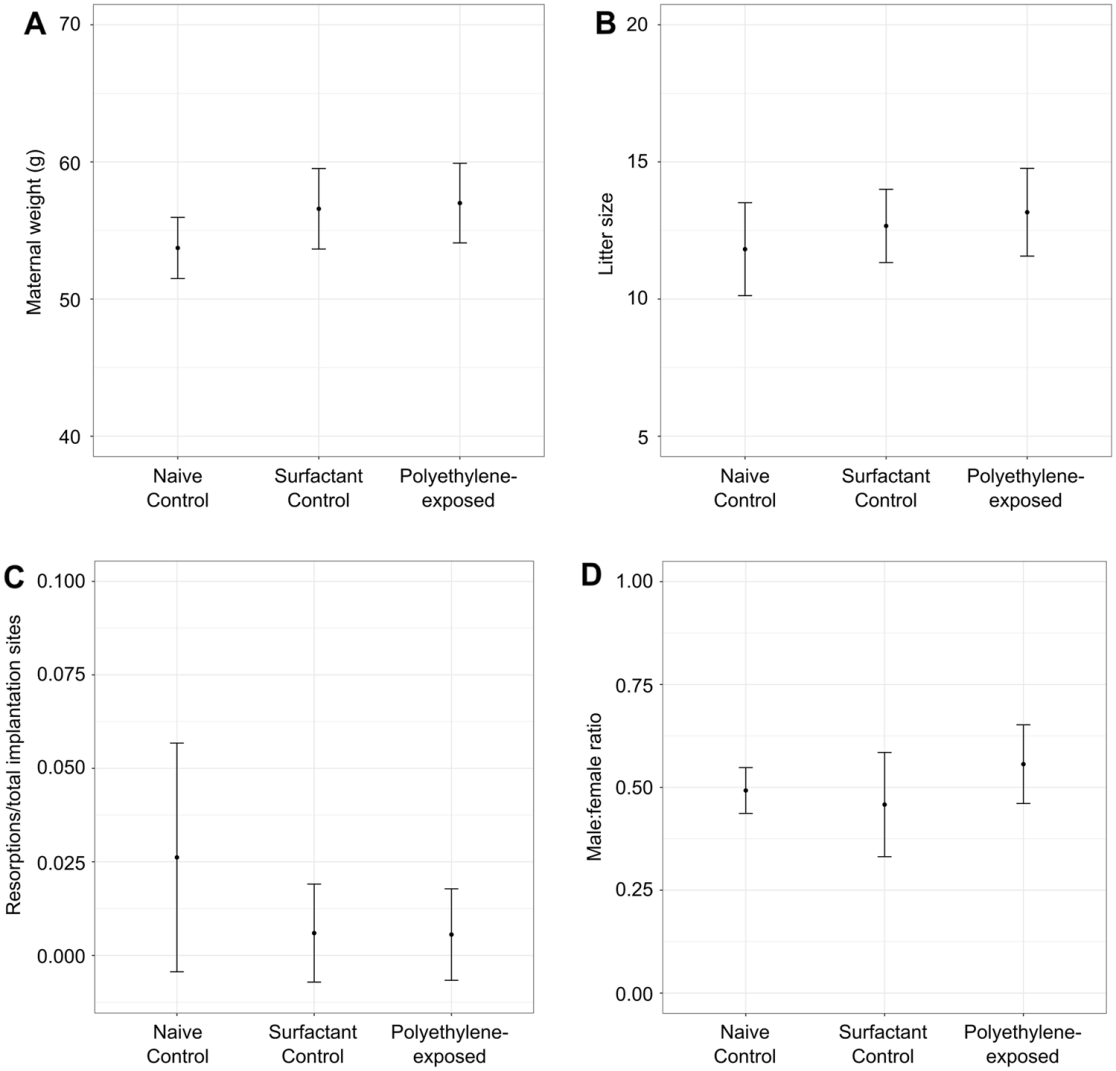
There was no effect of polyethylene exposure on (**A**) maternal weights, (**B**) litter size, (**C**) fetal resorptions, or (**D**) male:female ratio compared to controls at 17.5 days of gestation. *n* = 11–12 dams/group. For statistical analysis, the parameters were compared using a one-way ANOVA. Data are shown as means and 95% confidence intervals.

**Figure 2:**
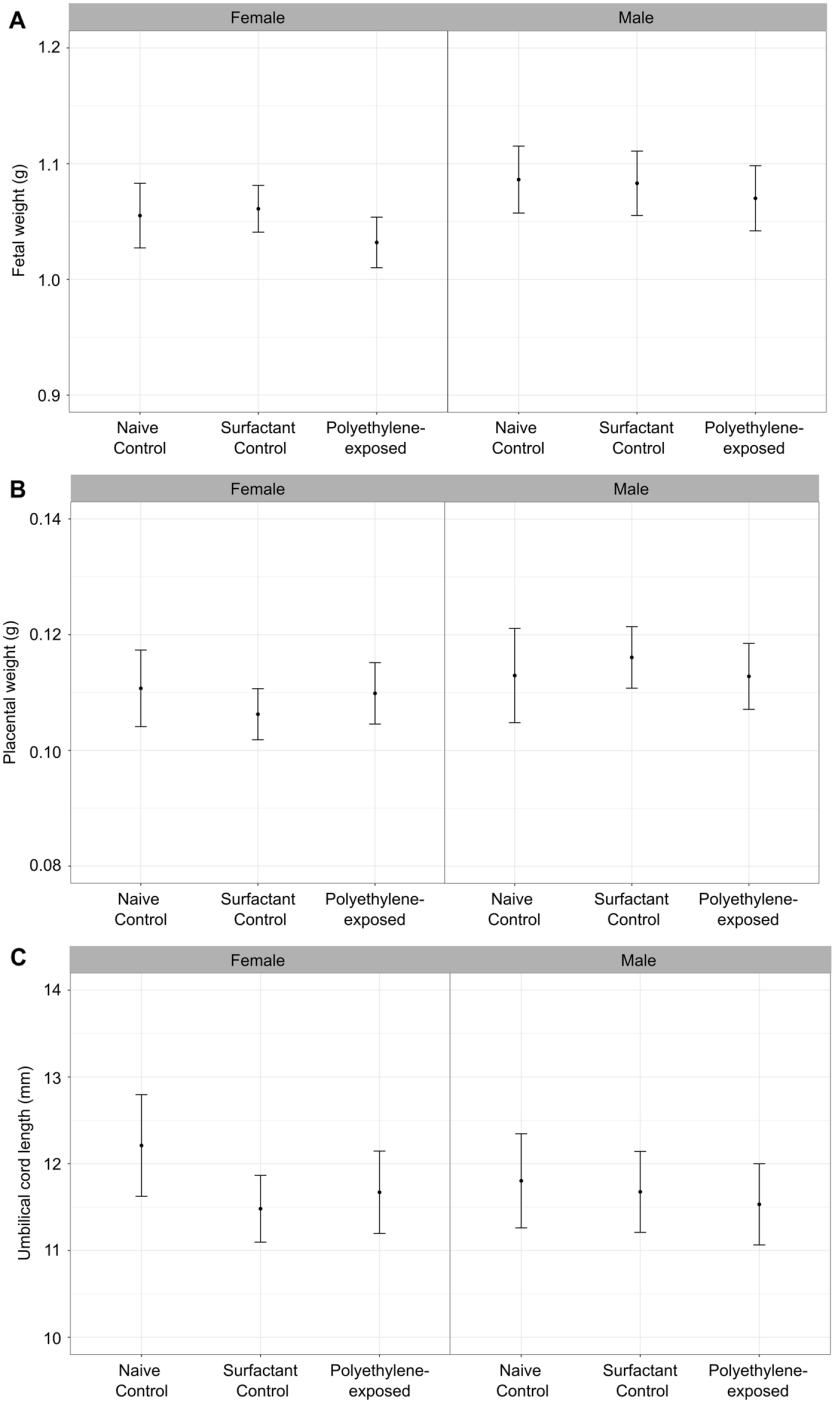
There was no evidence of growth restriction following maternal exposure to polyethylene micro- and nanoplastics. Fetal weights (**A**), placental weights (**B**), and umbilical cord lengths (**C**) in female and male fetuses at 17.5 days of gestation. For statistical analysis, the parameters were compared using a linear mixed effects model. *n* = 11–12 dams/group. Data are shown as means and 95% confidence intervals.

At embryonic day 17.5 (E17.5), there were no significant differences between groups in the maternal weights, litter sizes, number of resorptions or the male:female ratios (p > 0.05) (Fig. 1). The observed litter sizes were consistent with data previously reported for healthy CD-1 mice (~ 12 fetuses/litter)^23^. All of the groups consumed the same amount of water, an average of 8 (CI 7–9) mL per day (p > 0.05). While there was an effect of fetal sex on the fetal and placental weights (p < 0.05), there was no significant effect of group on the fetal weights, placental weights, or umbilical cord lengths (p > 0.05) (Fig. 2).

**Figure 3:**
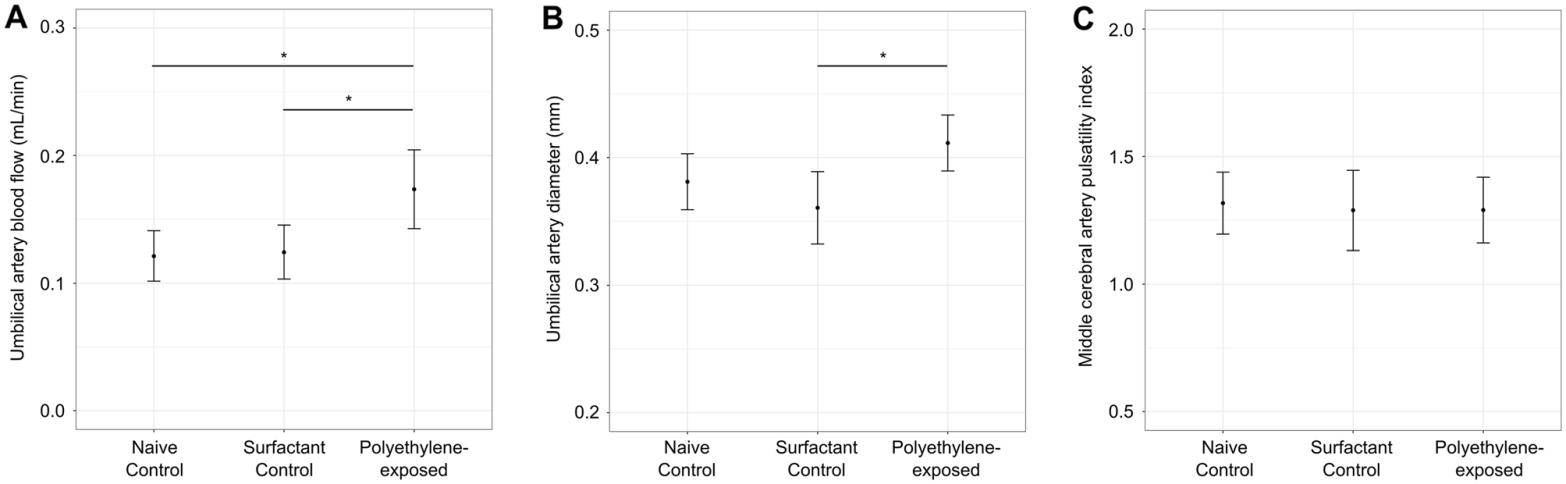
Impact of polyethylene micro- and nanoplastics exposure on placental and fetal hemodynamic responses at 17.5 days of gestation. Umbilical artery blood flow was significantly elevated in the polyethylene-exposed group compared to controls, *n* = 19–20 fetuses/group (**A**). Umbilical artery diameter was significantly increased in the polyethylene-exposed group compared to controls, *n* = 19–20 fetuses/group (**B**). There was no difference between groups for the middle cerebral artery pulsatility index. *n* = 10–18 fetuses/group (**C**). For statistical analysis, the parameters were compared using a linear mixed effects model. *p < 0.01. Data are shown as means and 95% confidence intervals.

In addition to exploring the effect of maternal polyethylene exposure on fetal and placental growth, we investigated the impact on fetal and placental hemodynamic responses using high-frequency ultrasound. Feto-placental blood flow was determined using the umbilical artery (UA) and fetal cerebral blood flow was determined using middle cerebral artery (MCA) blood velocity waveforms. The MCA pulsatility index (PI) is a measure of fetal brain sparing, where lower PI values indicate an adaptive response to fetal hypoxia that involves redistribution of oxygenated blood to the brain at the expense of other organs^24,25^. There was a significant effect of group on the UA blood flow (p < 0.01), with post hoc analysis showing a 43% increase in the polyethylene-exposed group (p < 0.01) compared to naive controls and a 40% increase compared to surfactant controls (p < 0.01) (Fig. 3a). There were no significant differences between the naive controls and the surfactant controls (p > 0.05). The increased UA blood flow in the polyethylene-exposed group was accompanied by vasodilation, with a 11% increase in the diameter of the UA (p < 0.01) compared to the control groups (Fig. 3b). The fetal heart rate was similar between groups (p > 0.05). There was no difference in the UA PI (p > 0.05), which is considered to be a surrogate measure of feto-placental vascular resistance^26^. Despite the significant impact on UA blood flow, there was no effect of group on measures of cerebral blood flow such as mean MCA velocity or MCA PI (Fig. 3c).

## Discussion

In this study, maternal polyethylene exposure throughout gestation did not significantly impact pregnancy outcomes or measures of fetal growth and development and no sex differences were observed between female and male fetuses in late gestation. Despite not observing any effect on fetal growth, polyethylene exposure resulted in increased UA blood flow compared to the control unexposed groups. For all of the parameters measured, there were no differences between the naive control and surfactant control groups. This illustrates that the increase in UA blood flow results from the polyethylene micro- and nanoplastics and not the surfactant additive. The increase in UA blood flow is most likely an adaptive response that increases oxygen extraction from the maternal blood to maintain fetal growth. This is likely a successful adaptive response since no late pregnancy fetal loss was observed, and no fetal cerebral vasodilatory response (lower MCA PI as found typically in human growth-restricted fetuses) was observed in the polyethylene-exposed fetuses. Therefore evoking the fetal brain sparing response was not required to protect the fetal brain from hypoxia in these deleterious circumstances.

A striking finding from this study was the absence of any effect of polyethylene on fetal growth or umbilical cord lengths. This result is consistent with Han et al. that reported no significant differences in neonatal weights following exposure of 10–45 μm polyethylene particles via intratracheal instillation at a similar concentration (6 μg/day)^27^. Compared to our previous study where we observed significantly decreased fetal weights and shortened umbilical cords following exposure to polystyrene micro- and nanoplastics^14^, the difference may be explained by a lower toxicity of polyethylene compared to polystyrene. In a study of the environmental and health hazards of 55 commonly used polymers, polyethylene was ranked as one of the least hazardous while polystyrene was identified as potentially carcinogenic and mutagenic^28^. Another explanation for the difference between polyethylene and polystyrene exposure studies is that the polyethylene exposure may only impact placental and fetal development during late gestation. For a noticeable effect on fetal weights, we expect that abnormal UA blood flow needs to occur over several days. Future studies will investigate UA blood flow earlier in gestation to determine when the onset of abnormal UA blood flow occurs following maternal exposure to polyethylene and polystyrene. While ultrasound provides information about the physiological responses following exposure, future studies will focus on histological assessment and stereology to determine the structural perturbations by which micro- and nanoplastics exposure might disturb placental development.

While the impact on fetal growth depends on the chemical composition of the plastic particles, the effect on UA blood flow was similar for 5 μm polystyrene particles and the polyethylene micro- and nanoplastics (average particle distribution of 1 μm) used in the present study (increase in blood flow of 48%^21^ compared to 43% in the present study). In contrast, exposure to 50 nm polystyrene nanoplastics resulted in a 25% decrease in UA blood flow^21^. This suggests the impact on placental function is primarily controlled by the size of the plastic particles.

This study has several limitations. First, the commercially available polyethylene particles used in this study ranged in diameter from 740 to 4990 nm, with most of the particles in the smaller size range. It is likely that some of the variability in our measurements is the result of this large particle distribution. We did not measure levels of micro- and nanoplastics in the maternal food which may be an important additional source of plastics exposure. Another limitation is that while isoflurane and oxygen levels were kept constant between groups, the ultrasound measurements do not reflect what we would expect for an awake animal. Finally, for technical reasons, the sex of the fetus could not be determined reliably by ultrasound. In the future, we plan to tag the fetuses with a dye injection^29^ so they can easily be identified upon dissection to collect samples for genotyping.

In summary, this study demonstrates that plastic polymer type is important when considering the impact on pregnancy and fetal outcomes. Maternal exposure to polyethylene micro- and nanoplastics throughout gestation resulted in abnormal UA blood flow but did not cause fetal growth restriction. While polystyrene appears to cause more significant deleterious effects on pregnancy, polyethylene still poses a risk for human health and further studies on exposure need to be performed in animal models and humans. Impaired UA blood flow is linked to many pregnancy complications with serious long-term consequences, highlighting the importance of developing regulations to minimize exposure to all types of micro- and nanoplastics during pregnancy and early life.

## Methods

### Animals

Thirty-five healthy adult CD-1 female mice purchased from Charles River Laboratories (St. Constant, QC, Canada) were used for this study. The females ranged from ages 6–18 weeks (average 11 (CI 10–12) weeks) and were bred in house (Health Sciences Animal Facility, Memorial University of Newfoundland, St. John’s, NL). Post breeding, the presence of a vaginal plug indicated the beginning of embryonic development and was assigned as embryonic day 0.5 (E0.5). At E0.5, dams were randomly assigned to one of three groups: control filtered drinking water (*n* = 11, referred to as *naive controls*), control 0.1% surfactant solution in filtered drinking water (*n* = 12, referred to as *surfactant controls*) or 10^6^ ng/L polyethylene micro- and nanoplastics (dissolved in 0.1% surfactant solution in filtered drinking water (*n* = 12)). Dams were singly housed and received their respective water from E0.5 to E17.5 (full-term in CD-1 mice is E18.5). The mice were given ad libitum access to food and water. The amount of water consumed was recorded. At E17.5, the dams were imaged using ultrasound biomicroscopy. Once images were collected, the dams were killed by cervical dislocation, the uterus was dissected, the umbilical cord length was measured, and the fetal and placental weights were recorded. Skin samples were collected for determination of fetal biological sex. All animal experiments performed were approved by Memorial University of Newfoundland Animal Care Committee (Animal Use Protocol 20-02-LC) and conducted in accordance with guidelines established by the Canadian Council of Animal Care. The study is reported in accordance with ARRIVE guidelines.

### Plastics exposure

The polyethylene drinking water solution was prepared using a biocompatible surfactant, necessary to coat the hydrophobic polyethylene particles prior to suspension in drinking water. Filtered drinking water was brought to a boil and a polyethylene sorbitol ester surfactant (Tween 80, Cospheric, Santa Barbara, California, USA) was slowly added to the water, cooled and then added on top of the polyethylene particles. The stock drinking solution contained 10^6^ ng of polyethylene spheres of size range 740–4990 nm (PENS-0.98, Cospheric, Santa Barbara, California, USA) and a 0.1% solution of Tween 80 in 1L of standard filtered water. The water was allowed to stir overnight to completely dissolve and disperse the particles. The hydrodynamic diameter of the polyethylene particles was determined using a ZetaSizer Nano-ZS system (Malvern Instruments). The average hydrodynamic radius was 1000 (CI 600–1400) nm. The concentration of the polyethylene particles was chosen based on previous work in our group using polystyrene, where a concentration of 10^6^ ng/L resulted in significant fetal growth restriction^14^. This concentration represents the upper end of concentrations of microplastics (> 1.0 × 10^6^ particles/L^30^) and nanoplastics (5.6 × 10^5^ ng/L^31^) reported in water sources.

### Ultrasound biomicroscopy

Using a high-frequency ultrasound system and a UHF57x transducer (centre frequency 40 MHz) (F2, VisualSonics, Toronto, Ontario, Canada), blood flow measurements were recorded at the umbilical artery (UA) and the middle cerebral artery (MCA) as detailed previously^32^. The UA blood flow reflects the total villous vascular volume and the MCA peak systolic velocity reflects fetal hemoglobin and arterial partial pressure of CO_2_. In each dam, two fetuses positioned in an optimal orientation were chosen for imaging. Dams were anesthetized with isoflurane in 100% O_2_ (4% for induction and 2% for maintenance). To ensure physiological conditions, maternal body temperature was maintained using a temperature regulated platform (35–37 °C). These parameters are best practices for measurements of physiology in experimental mice^33^. Temperature, heart rate and respiratory rate were monitored during imaging. Pulsed wave Doppler recordings were taken to measure blood velocity at a location where the smallest angle of insonation was found (always < 60° to allow for accurate angle correction). Diameter measurements of the UA were taken using motion-mode (M-mode) recordings at the same location as the Doppler image, with the transducer perpendicular to the vessel of interest. The intensity-weighted mean velocity of the Doppler waveform was traced as a function of time and used to measure the velocity–time integral (VTI). The UA vascular diameter was measured from the M-mode recording using the inner boundaries of the vessel walls and measuring the diameter during systolic and diastolic flow, defined by the speckle pattern of the arterial blood flow. Blood flow was calculated by multiplying the VTI by the vessel cross-sectional area and the fetal heart rate. The UA and MCA pulsatility indices (PI) were calculated as the difference between the peak systolic and end diastolic velocities, divided by the mean velocity over the fetal cardiac cycle. The UA PI is a measure of villous vascular resistance and the MCA PI reflects fetal brain sparing. All parameters were averaged over three fetal cardiac cycles.

### Fetal sex determination

The Sry primers used were: forward primer (CTCATCGGAGGGCTAAAGTG) and reverse primer (AAGCTTTGCTGGTTTTTGGA), product size 166 bp. Cyp24a1 primers were used as a DNA extraction quality control with forward primer (CCAAGTGTGCCATTCACAAC) and reverse primer (TCTCTCGCTGAACCTGGATT), product size 557 bp.

### Statistical analysis

All statistical tests were performed using the R statistical software package (www.r-project.org). All data are presented as means and 95% confidence intervals. Maternal weights, litter size, number of resorptions and fetal sex ratios were analyzed using a one-way analysis of variance (ANOVA) to evaluate the effect of exposure (naive control, surfactant control, polyethylene-exposed). For the fetal and placental weights and the umbilical cord lengths, a linear mixed effects model was used to account for measuring more than one fetus/placenta per dam since there are known similarities amongst littermates^34^. Exposure (naive control, surfactant control, polyethylene-exposed) and fetal sex (female, male) were the fixed effects and litter was treated as a random effect. For the ultrasound parameters (UA blood flow, UA diameter, UA PI, fetal heart rate, MCA velocity, MCA PI), a linear mixed effects model was used with exposure as the fixed effect and litter as a random effect. Statistical significance was defined as p < 0.05.

## Data Availability

Data from the current study are available from the corresponding author on reasonable request.
